# Challenges to ‘Last Mile’ Surveillance: Result of Programmatic Review of Integrated Skin NTDs Surveillance in Three Indonesian Districts

**DOI:** 10.3390/tropicalmed11050123

**Published:** 2026-05-06

**Authors:** Agrin Zauyani Putri, Ajib Diptyanusa, Regina Tiolina Sidjabat, Yatinawati Yatinawati, Yety Intarti, Irma Surya Kusuma, Khadijah Qurrata Ayun, Trijoko Yudopuspito, Muhammad Anwar Simanullang, Dwi Martanti, Achmad Naufal Azhari, Herdiana Herdiana, Yullita Evarini Yuzwar

**Affiliations:** 1 World Health Organization Indonesia Country Office, Jakarta 12940, Indonesia; agrinzauyani@gmail.com (A.Z.P.);; 2 Directorate of Communicable Diseases, Ministry of Health of the Republic of Indonesia, Jakarta 12950, Indonesia; 3 Global Institute for Disease Elimination, Abu Dhabi P.O. Box 764641, United Arab Emirates

**Keywords:** integrated surveillance, skin neglected tropical diseases, leprosy, lymphatic filariasis, yaws, Indonesia

## Abstract

Indonesia is approaching the ‘last mile’ of elimination for several skin-related neglected tropical diseases (skin NTDs): notably, leprosy, yaws and lymphatic filariasis (LF). However, persistent transmission in selected districts highlights systemic weaknesses in surveillance. This paper aimed to analyse the health system, operational and sociocultural barriers to integrated skin NTDs surveillance in Indonesia. A descriptive analysis of the national programmatic review of integrated skin NTDs was conducted in 2024, using a mixed-methods descriptive evaluation based on routine data and thematic analysis. Comparative case studies of the Belitung, Mimika and Sorong Selatan Districts were conducted using routine data, programme reports, and structured observations at primary health centres, district health offices and laboratories. Qualitative insights from programme managers, health workers and communities were thematically analysed. Integrated surveillance was constrained by fragmented governance, inflexible financing, and uneven workforce capacity, alongside operational challenges like delayed detection and geographic inaccessibility. Furthermore, sociocultural factors such as stigma and population mobility, combined with zoonotic LF transmission in Belitung, significantly undermine effectiveness and long-term programmatic sustainability. Despite strong national policy commitment and substantial progress in disease elimination, significant gaps remain between integration frameworks and operational realities at the district level. Accelerating skin NTDs elimination in Indonesia requires context-adapted integration, strengthened digital surveillance, sustained subnational financing, workforce capacity building and, in zoonotic settings, a One Health approach. Addressing these factors is essential for achieving and sustaining elimination in the last mile. Indonesia has achieved substantial progress across major skin NTDs, while also revealing persistent gaps that threaten the sustainability of elimination gains.

## 1. Introduction

Skin NTDs continue to pose a significant public health challenge in Indonesia, where leprosy, yaws and lymphatic filariasis (LF) remain persistently endemic [1,2]. Despite national progress in reducing the overall burden, the land of Papua consistently reports the highest prevalence of leprosy of 9.12 per 10,000 population, along with over 90% of probable yaws cases, and 26 districts showed ongoing LF transmission requiring mass drug administration (MDA) [3]. These patterns reflect inequities driven by geographic isolation, limited transport and health infrastructure, socioeconomic vulnerability and suboptimal sanitation [4,5]. Additionally, in other parts of Indonesia, remote communities often remain unreached by routine services, leading to delayed diagnosis, chronic disability and entrenched stigma that widen the gap between national elimination targets and local realities [6,7,8]. Because of these factors, Indonesia struggled to maintain its programmatic performance to achieve the ‘last mile’ of elimination.

Surveillance is central to interrupting the transmission of skin NTDs [9,10]; however, current systems in Indonesia face substantial operational constraints. Active case detection, contact tracing, laboratory confirmation and follow-up are inconsistently implemented, especially in remote areas with limited human resources and logistical capacity [8]. Underreporting remains common due to weak data flows, insufficient routine monitoring, fragmented information systems and limited supervision across decentralised health structures [1]. Additionally, frontline health workers reported transportation shortages and inadequate supplies. In resource-constrained settings, they also experienced limited training and challenges in applying standard diagnostic algorithms [11,12]. These gaps contribute to populations that remain undetected, misdiagnosed or untreated, thereby sustaining transmission cycles.

In response to these challenges, Indonesia is gradually shifting towards an integrated surveillance approach for skin NTDs, aligning with the World Health Organization (WHO) Skin NTD Strategic Framework [13]. This integration seeks to harmonise case detection, community outreach, laboratory processes and data reporting across leprosy, yaws and LF, recognising their overlapping geographic distribution, shared risk factors and similar operational needs. Experiences from Brazil, Liberia, Ghana, Nepal, Cameroon and several Pacific Island nations have demonstrated that joint community surveys, school-based screenings, mobile health worker networks and unified case management systems can significantly increase case detection and improve health system efficiency [14,15,16]. Nevertheless, integration must be adapted to Indonesia’s decentralised governance and the complex sociocultural landscape of many areas and diverse cultures.

While integrated skin NTD strategies are increasingly promoted in global policy frameworks, empirical documentation of integrated programme reviews at the national and subnational levels remains limited. Among the three diseases of interest, yaws is targeted for eradication, as humans are the primary reservoir, making biological eradication theoretically feasible. In contrast, leprosy and LF are targeted for elimination as a public health problem, rather than eradication, due to factors such as long incubation periods, subclinical infections, vector-borne transmission (for LF), and potential animal reservoirs. In Indonesia, elimination of leprosy and LF as public health problems is achievable and has progressed substantially, although subnational pockets of transmission remain. Yaws eradication is technically feasible but operationally challenging, particularly in remote and hard-to-reach endemic areas. This study contributes to the literature by systematically analysing cross-disease operational bottlenecks across leprosy, yaws and LF within a unified surveillance framework, offering practical insights for last-mile elimination settings. By identifying context-specific gaps and actionable opportunities, it outlines pathways to accelerate progress toward the sustainable elimination of skin NTDs in Indonesia.

## 2. Materials and Methods

### 2.1. Study Design

This study is a descriptive analysis of findings from the national programmatic review of integrated skin NTDs in 2024 by the Ministry of Health (MOH) of the Republic of Indonesia and the WHO. Leprosy, yaws and LF were selected as the main focuses, as these diseases are included within Indonesia’s national elimination targets and have established vertical control programmes under the Directorate-General of Disease Prevention in MOH. The review focused on surveillance performance, implementation practices and operational bottlenecks across selected districts with varying epidemiological and health system contexts.

### 2.2. Study Setting and Case Selection

Three districts were purposively selected to represent distinct surveillance challenges and to enable comparative analysis across different operational environments [17]. Selection criteria included: (i) persistent transmission or hotspot status for at least one of the three skin NTDs; (ii) differing geographic settings; (iii) variation in health system capacity and programme performance; and (iv) the feasibility of field assessment within the review timeline. Each district was treated as an individual “case”:

• Case 1: Belitung District was selected to reflect epidemiological surveillance challenges, particularly the presence of zoonotic LF and the need for enhanced entomological and clinical surveillance.
• Case 2: Mimika District was selected to illustrate urban and peri-urban surveillance complexities, including high population mobility, diverse health service providers, private sector involvement and security-related constraints that affect case detection and reporting.
• Case 3: Sorong Selatan District was selected to represent a remote and indigenous population context, characterised by geographic inaccessibility, cultural barriers, limited health workforce availability, and reliance on outreach-based surveillance.

These settings were chosen to capture heterogeneity in co-endemicity, health system capacities and sociocultural characteristics, thus allowing for a comprehensive understanding of the operational realities of integrated skin NTDs surveillance in Indonesia.

Case definitions followed national MOH guidelines aligned with WHO standards:

• Leprosy cases were defined clinically, based on the presence of characteristic skin lesions with sensory loss and/or peripheral nerve involvement, supported by slit-skin smear microscopy where available.
• Lymphatic filariasis infection was assessed through antigen or antibody rapid diagnostic tests and/or microfilaria detection via a thick blood smear, depending on endemic species. Chronic cases were defined clinically as lymphoedema or hydrocele, consistent with filarial pathology.
• Yaws cases were identified through the clinical presentation of characteristic lesions and confirmed serologically using treponemal and non-treponemal tests, when available.

### 2.3. Data Collection

Data collection consisted of two complementary components. First, existing aggregate programme data were reviewed from surveillance databases, district-level annual reports, treatment registers, laboratory logs and supervisory records to assess progress against national indicators for the elimination of leprosy, yaws and LF. The inclusion criteria for data sources were: (i) official national or provincial reporting systems; (ii) WHO-validated monitoring data; and (iii) peer-reviewed publications, where available. Second, data were extracted from the 2024 national programmatic review of integrated skin NTDs in Indonesia. This included structured observations conducted at community health centres, district health offices (DHOs) and district laboratories, using predefined criteria and standardised checklists to assess the availability of surveillance tools, diagnostic capacity, reporting flows, integration of programme activities and adherence to national guidelines. Additionally, case studies involving district programme managers, clinicians, laboratory staff, frontline health workers and community members were analysed to capture perceived enablers and barriers, community engagement and contextual factors influencing service delivery.

Data were triangulated across sources where possible. Findings from desk review were cross-checked during field visits through interviews with district health officers, laboratory personnel, and programme managers. Discrepancies were discussed further with the field office to ensure internal validation.

### 2.4. Data Analysis

The review team consisted of national and international public health professionals with expertise in NTDs, health systems, and programme implementation. Several members had prior involvement in policy advisory roles related to NTD elimination in Indonesia. To mitigate potential bias, the analysis incorporated multiple data sources, triangulation of documentary and field data, and independent coding by two reviewers (A.D., A.Z.P.). Interpretations were discussed within the multidisciplinary team (R.T.S., Y.I., T.Y., Y.E.Y., H.H.) to ensure a balanced representation of programme strengths and challenges. Differences in interpretation were resolved through consensus during structured analytical meetings, with reference to source documentation to ensure consistency and transparency.

Data were analysed descriptively. Quantitative programme indicators were summarised to assess district performance, relative to national elimination benchmarks [3]. Qualitative findings from structured observations and case studies were analysed thematically to identify recurring patterns related to surveillance implementation, including enablers, barriers and district-specific operational constraints [18]. Themes were synthesised across the three cases to highlight shared challenges as well as context-specific considerations, adapted from the framework for the integrated control and management of skin NTDs by the WHO (i.e., surveillance, case detection, morbidity management, laboratory capacity, financing, and governance) [13]. Initial coding domains were developed deductively based on these predefined frameworks, with inductive identification of emerging themes from field observations and document review. Longitudinal indicators of disease elimination were reviewed to contextualise district-level findings and to inform the interpretation of surveillance bottlenecks identified during thematic analysis. The findings are presented narratively to illustrate how epidemiological, sociocultural, and health system contexts influence the integrated skin NTDs surveillance outcomes.

### 2.5. Ethical Considerations

Ethical review was waived for this work. The analysis presented in this manuscript is based solely on the descriptive review of programmatic data and routine implementation findings. The national programmatic review of integrated skin NTDs was conducted as part of the mandated programme activities by the Ministry of Health, rather than as a research study. The data utilisation was approved by the Ministry of Health prior to analysis (Ref. No. PM.03.03/C.III/3440/2025).

## 3. Results

**Figure 1:**
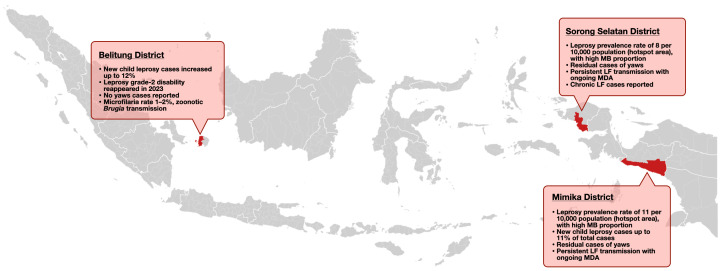
Geographic location and epidemiological profile of three skin NTD case study districts included in the 2024 integrated review.

The case study was performed in Belitung District (Bangka Belitung Islands Province), Sorong Selatan District (Southwest Papua Province), and Mimika District (Central Papua Province), as shown in Figure 1. The programmatic review of the three districts revealed that a complex interplay of different context-specific gaps constrains the implementation of integrated surveillance for skin NTDs. These findings are categorised into three major themes of challenges: health systems, socio-cultural barriers, and operational and epidemiological complexities.

### 3.1. Progress of Skin NTDs Elimination in Indonesia

**Figure 2:**
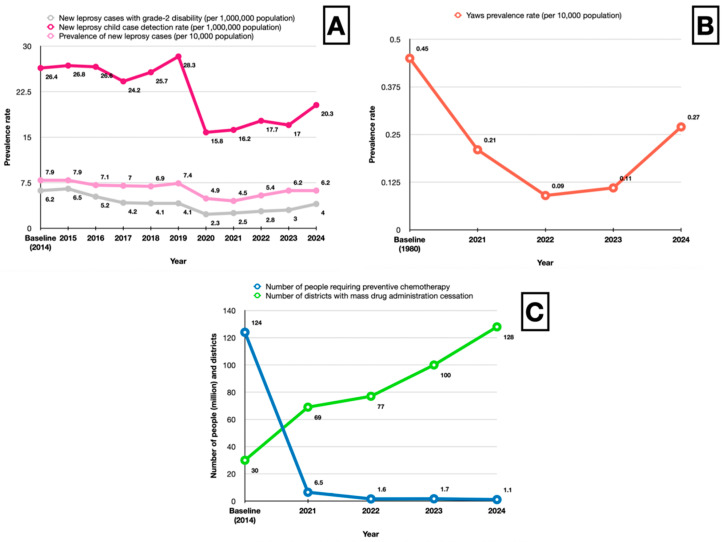
National trends in leprosy (**A**), yaws (**B**), and LF (**C**) disease elimination indicators in Indonesia.

Indonesia has achieved substantial progress across major skin NTDs, while also revealing persistent gaps that threaten the sustainability of elimination gains (Figure 2).

For leprosy, the national prevalence has declined markedly compared with the early 2000s. Recent trends show increases in child case detection, which may be attributable to strengthened active case finding following disruptions during the COVID-19 pandemic, leading to improved detection of previously missed cases. However, the simultaneous rise in grade 2 disability strongly suggests delayed diagnosis, as this indicator reflects late presentation and a prolonged untreated infection. The observed increase in reported cases from 2019 to 2024 was due to improved case finding activities. Yaws has been reduced to very low levels nationally after the initiation of the Treponema Control Programme Simplified (TCPS), with cases now confined to limited geographic foci with a very low prevalence rate for children aged 1 to 15 years (0.27 per 100,000 population). For LF, the population requiring preventive chemotherapy has decreased about 98% since the scale-up of MDA in 2014, leaving only 108 out of 236 endemic districts waiting for post-MDA evaluation. Overall, Table 1 and Supplementary Table S1 provide longitudinal evidence of progress and residual vulnerabilities in Indonesia’s skin NTD elimination efforts.

### 3.2. Health System Limitations: Governance and Finance

The review highlights the fragmentation of Indonesia’s decentralised health system. The health system capacity in Indonesia is strong, with over 55% of the community health centres having adopted the integrated primary healthcare approach, with the availability of special allocation funds to support public health activities. However, the operational reality often contradicts this potential. Districts continue to rely on central government financing, which limits the districts’ capacity to strategically allocate funds based on local needs. The findings indicate that, in practice, programmes for communicable diseases such as HIV, malaria and tuberculosis, as well as non-communicable disease (NCD) programmes including stunting prevention and maternal and child health, often displace funding for NTDs. In Sorong Selatan, accessing district-level funds for LF activities was described as “difficult, even impossible,” because the disease is not prioritised in local development plans and often relies on limited community health centre operational funds for surveillance activities. However, in Mimika District, there are opportunities, with the presence of the mining industry, where public–private cooperation for health care is strong. This variability of political commitment and financial resources is critical to ensure continued commitment. Additionally, the recent reassignment of regional public health laboratories has disrupted the detection leprosy and yaws, as well as LF evaluation surveys, with only 20% of the necessary surveys completed in 2024.

This fragmentation extends to human resources and technical capacity. Indonesia has a strong public health laboratory system, equipped with laboratory quality and skilled staff from national, regional, provincial, district, and community health centre levels. However, specific technical proficiency for skin NTDs surveillance and disability management is lacking. In Mimika District, laboratory technicians at the community health centre level have not been trained in identifying LF microfilaria, although the laboratory infrastructure is adequate for malaria microscopy and is thus sufficient for detecting LF microfilaria. This gap prevents the integration of LF and malaria routine surveillance. The programmatic review also identified insufficient clinical training in Sorong Selatan, where health workers reported difficulties in differentiating between Paucibacillary (PB) and Multibacillary (MB) leprosy cases, and no formal refresher training had been conducted in recent years. Additionally, they struggled to refer patients to dermatologists with leprosy reactions due to the low number of dermatologists in the area. This highlights two aspects in which expertise might be insufficient: health workers may not possess the necessary knowledge, or the specialised medical experts might not be available. In Belitung District, only one laboratory technician was available at the district level with lack of formal training, leading to sub-optimal active case finding for conditions like lymphedema, leprosy, and yaws. Overall, among the 52 formal health facilities in these three districts, approximately 90% of health professionals have never received formal training in the diagnosis and case management of leprosy, yaws, or LF.

### 3.3. Operational Barriers: Data, Logistics, and Access

The implementation of the integrated surveillance is hampered by gaps in information systems, rigid supply chain processes, and suboptimal referral linkages. Although Indonesia has launched SatuSehat, a national digital health platform intended to unify all health data, NTD indicators have not yet been integrated. Therefore, in all districts, the recording and reporting of skin NTDs remain paper based. The programme manager at DHO is responsible for filling out the monthly records and reports, based on manual reports from every community health centre. Afterwards, the data will be reported to the national programme using an Excel form, reporting cases on a per disease basis. In general, routine data were manually compiled at the district level, increasing the risk of reporting errors and delays. The Ministry of Health has developed a new recording and reporting system for both leprosy and yaws called SITASIA. However, it is not yet fully functional, and programme managers continue to report using Excel formats. Continued reliance on paper-based reporting leads to delays in case detection and treatment, slows data aggregation and often results in incomplete datasets. In Mimika, poor linkage between hospitals and community health centres leads to unrecognised community-detected leprosy cases, patient loss within the surveillance system and missed referrals for disability care and follow-up, despite the availability of social welfare support for people with leprosy and chronic LF-related disabilities. Additionally, no referrals observed for disability management required hospital-level care in the three observed districts.

Logistical challenges were also identified across leprosy and yaws programmes in the three districts. It affects surveillance implementation and treatment availability. For leprosy, a local operational protocol for treatment exists, but the available multi-drug therapy (MDT) at the community health centre is only for patients who have already registered and started treatment. While intended to ensure treatment completion, this protocol creates a barrier for newly detected cases. Consequently, data-reporting to the national programme was hindered by an insufficient MDT supply. The case studies revealed that the detection-to-treatment delay ranged from one week to three months. In the yaws programme, the diagnostic capacity remained constrained due to the siloed use of resources. Although serological tests such as RPR and TPHA were available for the syphilis programme, they were rarely utilised for yaws diagnostics. Instead, treponemal rapid diagnostic tests (RDT) were used for clinical confirmation. This depended on test availability, further complicating case verification. Additionally, inconsistent stocks of azithromycin challenged the adherence to standard treatment guidelines.

Finally, geographical barriers undermine the operational effectiveness and surveillance coverage of skin NTDs programmes in eastern Indonesia. In Sorong Selatan, it may take between 6 and 12 h to reach remote villages. This not only adds higher costs but also complicates logistics. Meanwhile, Mimika faces security concerns and extreme highland terrain that prevent routine services from reaching communities. Furthermore, transport and financial limitations hinder access for people with disabilities. Although mobile health centres are present, they primarily reach mothers and those at home, missing men and school-aged children who are absent from home during the day.

### 3.4. Sociocultural and Epidemiological Challenges

Indonesia’s socio-cultural landscape is defined by its diversity and shaped by a multicultural and multi-religious society. Three selected districts showed different dynamics in stigma, health literacy, and community engagement. Community-level stigma was identified as one of the barriers to the elimination of skin NTDs, particularly in Papua. In some communities, lower stigma for leprosy was noted, linked to cultural practices regarding grief and finger cutting. In contrast, the LF programme in Papua faces resistance, with ongoing MDA activities encountering fear and mistrust towards health authorities, which were exacerbated by community experiences during the COVID-19 pandemic. Furthermore, there are many hard-to-reach communities in coastal and highland areas, with different ethnic tribes having unique characteristics. These factors impede community outreach and service delivery. In contrast, leprosy contact screening in Belitung remains limited to immediate family members to prevent the stigma associated with screening other social contacts. Yet, opportunities exist within their protocol for screening leprosy contacts during other health screenings, allowing for integration with other health activities without triggering potential social stigma.

This landscape is further complicated by epidemiological complexity, particularly the evidence of zoonotic lymphatic filariasis transmission in Belitung. Although the district received a national elimination certificate in 2016 for achieving lymphatic filariasis elimination as a public health problem, subsequent investigations in 2020 and 2024 have demonstrated persistent *Brugia malayi* infection in animal reservoirs, including domestic and peridomestic mammals, indicating ongoing zoonotic transmission. These studies suggest that animal hosts may act as a sustained source of infection for mosquito vectors, enabling continued transmission even in the absence of detectable human cases and creating conditions for recrudescence. The presence of competent vectors, combined with close human–animal–environment interactions, undermines the long-term durability of the elimination gains and highlights the limitations of surveillance systems that focus exclusively on human infection. In such contexts, transmission may continue at low levels despite meeting the human-based elimination criteria. This biological complexity may contribute to recrudescence, even after districts have passed Transmission Assessment Surveys (TAS) and received an elimination certification; hence, there are discrepancies between national elimination indicators and district-level recrudescence. Together, this evidence underscores the need for post-elimination surveillance strategies in Belitung to incorporate animal and entomological components within a One Health framework to prevent the re-establishment of transmission. Table 2 demonstrates the salient findings of the 2024 Skin NTDs Programme Review in Indonesia.

## 4. Discussion

This paper underscores that despite nearing the last mile in eliminating LF, leprosy, and yaws, Indonesia faces complex system challenges. While Indonesia is among the countries that adopt and implement the WHO strategic framework for the integrated control of skin NTDs [19], the results from the programmatic review in the Mimika, Sorong Selatan, and Belitung Districts show an apparent gap between policy intent and operational realities. The key programme gaps identified are inconsistencies in case detection and delayed treatment initiation, limited technical and clinical capacity at district level, fragmented surveillance and predominantly manual reporting systems, dependence on centralised financing with constrained district-level autonomy, and insufficient integration of post-elimination and zoonotic surveillance strategies. These findings align with previous analyses, indicating that NTD surveillance in Indonesia remains challenged by geographic fragmentation, decentralised health governance, and uneven technical capacity across districts [1,2]. Addressing these gaps requires a shift from a disease-specific approach to a more integrated model that leverages the existing health infrastructure and systems [20].

The WHO defines integration as the simultaneous implementation of two or more programme activities through joint delivery of interventions at the community and/or health facility levels [13,19]. Our findings indicate that while yaws surveillance has been incorporated into outreach activities at the community health centre, particularly through maternal and child health programmes and village-level screening, integrated surveillance remains suboptimal. The research suggests three primary domains for optimising this integration: (1) across disease programmes, (2) into routine primary care, and (3) into health education [21,22,23]. Several studies also highlight that the skin NTDs integration into existing platforms has the potential to improve efficiency, cost-effectiveness, and service delivery [20,24,25]. Since leprosy and tuberculosis share mycobacterial origins and require rigorous contact tracing, leprosy screening could be systematically integrated into active TB case finding activities [26]. Similarly, LF and malaria, as vector-borne diseases, share common operational ground; synergies could be achieved through coordinated surveys [27]. Furthermore, school health programmes offer a platform to deliver preventive chemotherapy (PC) medicines and conduct skin screening for school-aged children simultaneously [9,28]. Beyond surveillance, there is also a critical need for integrated morbidity management and disability prevention (MMDP). Developing a comprehensive care package—for example, combining lymphedema management, leprosy wound care, and diabetic foot care—could reduce stigma and improve efficiency compared to vertical disease-specific clinics [29]. Additionally, the use of teledermatology and digital health tools, including mobile applications, may act as potential enablers of integrated skin NTD surveillance in remote settings [30,31].

However, for integration to be effective, critical enabling factors must be present, including adequate funding, strong multilevel governance, health worker motivation, and active community engagement [21]. Although the overall health system capacity in Indonesia is strong, resources dedicated to the implementation of integrated surveillance for skin NTDs remain inconsistent, limited, and subject to organisational and geographical constraints. The health sector functions within a decentralised system [32]; following Law No. 22 of 1999, the district level assumed the responsibility to provide public services, including health [33]. Yet, districts continue to rely on central government financing, which limits their capacity to strategically allocate funds based on local needs [8]. While integrated surveillance, digital tools, and One Health approaches offer conceptual advantages, their implementation also requires the consideration of resource availability, workforce capacity, and decentralised governance structures [34]. In Indonesia’s district-based health system, feasibility may vary substantially across provinces, depending on fiscal space, digital infrastructure, and intersectoral coordination capacity [35]. Raising the visibility of skin NTDs through advocacy encourages sustainable financial commitment and generates political support, particularly to strengthen health care systems at the sub-national levels [36]. Government commitment must be operationalised by establishing skin NTDs elimination as a national indicator and integrating it into the national health development plan [37]; this enables regional authorities to translate these national goals into their own local development strategies. Consequently, political commitment is essential for the ensured continuous effort throughout both the pre- and post-elimination phases. Political will is critical to guarantee the funding, for example, in post-validation surveillance (PVS) for LF. A study in Niue identifies a strategic opportunity to integrate LF PVS with the WHO STEPwise approach to NCD risk factor surveillance (STEPS), allowing for sustained periodic PVS to ensure no re-establishment of LF transmission, even within a limited funding environment [38].

The health workforce in Indonesia is characterised by inadequate and uneven distribution, with disparities more pronounced at the district level than the provincial level [39]. These disparities are particularly acute in the Papua region, where service delivery is impeded by difficult terrain and further complicated by high rates of workforce turnover [40,41]. This high turnover creates a knowledge drain, which results in new personnel being appointed to new NTD roles without the required knowledge and skills. To mitigate these gaps, the development of a unified training curriculum is a vital aspect for the success of integrated programmes. In co-endemic areas, training modules must be consolidated, ensuring that health workers receive simultaneous surveillance training for all relevant skin NTDs, rather than fragmented, disease-specific instruction [10,11]. A significant opportunity exists to leverage Plataran Sehat, the MOH of Indonesia’s digital learning platform. By utilising this platform to disseminate standardised curricula and modules, Indonesia can systematically address the training deficits in health workers and ensure consistent competency standards across diverse regions. However, the platform’s reliance on stable internet connectivity presents a critical limitation for remote areas, particularly in Eastern Indonesia. This infrastructure gap is substantial; a study by Aisyah et al. highlights that 38.31% of community health centres in the Maluku and Papua islands lack internet access [42]. Consequently, digital implementation in regions like Papua must be complemented by targeted in-person training to ensure that workforce capacity building is not hindered by infrastructure constraints [43]. Beyond technical proficiency, capacity building must also encompass leadership development. Evidence indicates that supportive supervision and active engagement with health workers at the peripheral level positively affect the effectiveness of disease monitoring and reporting in surveillance activities [44,45,46]. Geographical barriers in Indonesia contribute to the persistence of ‘never-treated populations’, referring to remote communities that are consistently missed by preventive chemotherapy for LF and yaws. In these settings, the role of community health workers (CHWs) is critical for bridging the last-mile gap; therefore, training initiatives must explicitly extend to CHWs in hard-to-reach areas [47,48]. CHWs not only support routine health services but also raise awareness within communities, which is essential for programme acceptance [49,50]. Leveraging local trusted figures such as religious leaders, traditional healers, and other representatives, for example, from women’s groups, has been shown to enhance community trust and participation in NTD elimination programmes [50,51].

Operational capacity is further challenged by the surveillance infrastructure. Based on our findings, the monitoring and reporting of skin NTDs remain paper based. Although the national programme has initiated a health information management system, it remains non-operational, leaving programme managers to rely on old formats. Findings from studies in Kenya indicate that multiple reporting channels contribute to inconsistent and inaccurate surveillance reports [45,52]. This is consistent with our findings: the current variety of reporting channels for skin NTDs in Indonesia is prone to data inaccuracies and backlogs [53]. Therefore, for integrated surveillance to succeed, existing vertical NTD programmes must first adopt unified reporting mechanisms [24]. While the introduction of SITASIA, a new electronic reporting system for leprosy and yaws, aims to minimise these discrepancies across surveillance levels, its introduction should be accompanied by adequate training for personnel [53]. Furthermore, infrastructural barriers such as limited internet access pose a significant risk to report submission [54]. This is particularly critical, given that the burden of NTDs is disproportionately concentrated in Eastern Indonesia [2,41], a region characterised by gaps in regional development and connectivity compared to other regions. In alignment with Indonesia’s digital health transformation to tackle health data fragmentation caused by the high number of health applications [55], it is recommended that skin NTDs indicators be integrated into the national SatuSehat platform [56]. This is to ensure long-term standardisation and accessibility. By connecting services between hospitals, community health centres, and laboratories, SatuSehat presents a significant opportunity to address the logistical hurdles of skin NTDs management and to streamline the referral for disability care.

Finally, specific challenges remain regarding the biological complexity of elimination. Several countries in Asia have reported the presence of zoonotic *Brugia* infection in cats, dogs, and monkeys [57,58,59,60]. Given that *Brugia* species are the major cause of LF in Indonesia, this poses a significant challenge. *B. malayi* is endemic in Belitung. The district, however, passed three TAS in 2016, leading the MOH to grant the elimination certificate in 2017 [61]. However, a subsequent study in 2021 detected *B. malayi* microfilaria (Mf) prevalence between 1.7% and 5.9% in five villages in Belitung District, necessitating the MDA using the alternative regimen with Ivermectin, Diethylcarbamazine (DEC), and Albendazole [62]. This evidence indicates that the existing surveillance protocols are insufficient in settings with zoonotic reservoirs. In Belitung District, programme assessments should be distinguished by two evaluation units (EUs), with microfilaria prevalence assessed separately in urban areas and in five villages where zoonotic transmission is suspected. In the EU, comprising five villages with potential zoonotic transmission, MDA must be continued in line with the current WHO recommendation. In parallel, One Health frameworks must be urgently established, incorporating surveillance and intervention targeting animal reservoirs to prevent recrudescence [60,63].

This study has several limitations. First, findings were derived from routine programme data and structured district-level review documentation, which may be subject to reporting inconsistencies and variable data completeness across districts. Some districts relied on manual reporting systems, increasing the risk of transcription errors and delays in data consolidation. Second, observations regarding technical and operational constraints were based on documented programme review inputs and may not capture all contextual nuances. Third, the analysis was descriptive and did not include primary qualitative data collection or independent verification of district-level reports.

Despite these limitations, the study has important strengths. It draws upon the most recent national and district-level programme data, providing an updated overview of the elimination progress across leprosy, yaws and LF. The analysis integrates multiple data sources, enabling insights into epidemiological trends and operational barriers, and demonstrating how shared systemic constraints, rather than disease-specific weaknesses alone, shape elimination trajectories. By examining skin NTDs within a single analytical framework, this study offers a systems-level perspective on shared bottlenecks in the ‘last mile’ of elimination, particularly practical values for policy refinement and programme planning in the context of the 2030 elimination targets. Future programme development would benefit from patient–public involvement and community engagement to better understand barriers to early care-seeking, stigma reduction, and treatment adherence. Dedicated qualitative or participatory studies could further explore patient perspectives and inform context-sensitive implementation strategies.

## 5. Conclusions

The programme review and study findings suggest that achieving the final stages of skin NTDs elimination in Indonesia does not solely depend on disease-specific progress but on the strength and adaptability of integrated surveillance systems. While integration offers clear opportunities to improve efficiency and reach underserved populations, the programme review highlights persistent health system fragmentation, operational constraints and sociocultural barriers that undermine last-mile performance. Sustained political and financial commitment at subnational levels, harmonised digital surveillance, integrated training for frontline health workers and context-sensitive community engagement are critical to sustain gains. Furthermore, the challenge of zoonotic transmission requires a shift in policy. Elimination strategies must be complemented by the development of international guidelines for zoonotic LF surveillance, filling the current gap in the global health framework. Future efforts must operationalise a One Health approach to prevent the re-establishment of transmission. Further research is needed to assess the readiness of health facilities in Indonesia to deliver integrated skin NTDs services, including service availability, workforce capacity and diagnostic capability.

## Figures and Tables

**Table 1:** National elimination indicators and progress trends for leprosy, yaws and LF in Indonesia, including baseline values and most recent reported data.

Disease	Indicators	Baseline (2014)	Recent Progress (2024)
Leprosy	New leprosy cases with grade 2 disability (rate per 1,000,000 population)	6.2 per 1,000,000	4.0 per 1,000,000
	New leprosy child case (<15 years) detection rate per 1,000,000 population	26.4 per 1,000,000 (17,025 new child cases)	20.3 per 1,000,000 (1420 new child cases)
	Number of new leprosy cases annually	17,025 new cases (prevalence of 0.79 per 10,000 population)	14,698 new cases (prevalence of 0.62 per 10,000 population)
Yaws	Interruption of transmission for 3 consecutive years	Prevalence rate of 0.45 per 100,000 population (in 1980)	Prevalence rate of 0.27 per 100,000 population
LF	Population requiring preventive chemotherapy (PC) for LF	124 million people	1,093,700 people
	Elimination as a public health problem through sustained infection below transmission assessment survey (TAS) thresholds for at least 4 years after stopping MDA	30 districts out of 236 endemic districts (12.7%)	128 districts out of 236 endemic districts (54.2%)

**Table 2:** Thematic summary of health system, operational, and sociocultural findings from the 2024 national programme review across three selected districts.

Category	LF	Leprosy	Yaws
Funding	• Regional Public Health laboratory restructuring created uncertainty in coordination and funding for LF surveys. • In Belitung, no DHO or sector budget supported case finding, training, or programme activities.	• MDT supply at community health centres was limited to registered patients; newly detected cases could not start treatment immediately.	• Limited availability of TPHA/RPR; tests were often prioritised for syphilis. • Inconsistent azithromycin supplies affected adherence to treatment guidelines. • Use of treponemal RDTs depended on stock availability.
Training	• Training varied; direct observation for treatment was not uniformly applied during MDA. • No formal training for lymphoedema care in Belitung. • Regional/provincial labs demonstrated good microfilaria diagnostic capacity.	• Staff understood diagnostic criteria but lacked training in wound care and disability management. • Only one laboratory technician in Belitung; no recent refresher training.	• Limited training in clinical diagnosis of yaws. • Training was disease-specific and reached few community health centres.
Data Reporting	• MMDP indicators were reported annually without integration with HMIS. • Routine data lacked age and sex disaggregation. • Hydrocele data are absent, preventing accurate prevalence estimation.	• Reporting was completed manually, using patient cards. • Detection-to-treatment delay ranged from 1 week to 3 months. • School surveys are informal, generating incomplete coverage data.	• Manual reporting from the community health centres to DHO; monthly compilation by programme managers. • The reporting system (SITASIA) was under development but not fully operational.
Referral System	• No referrals observed for disability management requiring hospital-level care.	• No referrals observed for disability management requiring hospital-level care.	• No referrals observed for disability management requiring hospital-level care.
Logistics	• Quality concerns regarding *Brugia* rapid tests and transition from TAS to *Brugia* Impact Survey (BIS) caused survey delays and backlogs.	• Policy restricted MDT stocks to existing patients; newly detected cases did not receive immediate treatment.	• Limited and inconsistent supplies of serological tests and azithromycin constrained diagnosis and treatment.
Stigma and Acceptance	• COVID-19 response reduced MDA uptake and increased mistrust. • Communities were confused when MDA resumed after recrudescence.	• Stigma hindered the implementation of single-dose rifampicin post-exposure prophylaxis (SDR-PEP) and contact examination in some areas. • In Papua, stigma was lower due to cultural norms. • In Belitung, contact screening was limited to household members.	• Low awareness of MDA reduced participation and community engagement.
Zoonotic Issues	• Zoonotic filariasis posed a significant epidemiological barrier to LF elimination.	• Not identified.	• Not identified.
Existing Integration	• Community outreach platforms supported LF MDA.	• Integrated lymphoedema–leprosy management piloted in two districts in Papua (Jayapura and Manokwari).	• SITASIA was used for leprosy and yaws reporting.
Opportunities	• Strong health system structures enable integration of MMDP with leprosy disability services. • Social welfare programmes offer long-term support for people with disabilities. • Engagement with private industry and religious, women’s, or youth groups is possible. • Additional funding available in Papua.	• Opportunities existed to integrate case finding, surveillance, training, and community engagement.	• Strong relationships with schools, religious groups, and private sector supported community mobilisation for MDA and screening.
Suggestions for Integration	• Introduce integrated skin NTD training for community health centre and DHO staff. • Integrate yaws diagnostic and treatment logistics with STI/MDA programmes for supply consistency. • Develop integrated surveillance standard operating procedures. • Strengthen disability care platforms across skin NTDs. • Develop unified training curricula for skin NTDs. • Develop technical guidelines on school-based NTD programmes. • Utilise teledermatology in remote settings. • Engage traditional leaders and CHWs to reduce stigma and improve trust. • Establish collaboration policies with the animal health sector to monitor LF reservoirs.		

## Data Availability

The original contributions presented in this study are included in the article/Supplementary Materials. Further inquiries can be directed to the corresponding author.

## Supplementary Materials

The following supporting information can be downloaded at: https://www.mdpi.com/article/10.3390/tropicalmed11050123/s1, Table S1: Detailed disease elimination targets and progresses of Indonesia.

## Abbreviations

The following abbreviations are used in this manuscript:

BIS	*Brugia* Impact Survey
CHW	Community health worker
COVID-19	Coronavirus disease-19
DEC	Diethylcarbamazine
DHO	District Health Office
EU	Evaluation unit
LF	Lymphatic filariasis
MB	Multibacillary
MDA	Mass drug administration
MDT	Multi-drug therapy
MMDP	Morbidity management and disability prevention
MOH	Ministry of Health
NCD	Non-communicable disease
NTD	Neglected tropical disease
PB	Paucibacillary
PC	Preventive chemotherapy
PVS	Post-validation surveillance
RDT	Rapid diagnostic test
RPR	Rapid plasma reagin
SDR-PEP	Single-dose rifampicin post-exposure prophylaxis
SITASIA	*Sistem Pendataan Kusta dan Frambusia* (Leprosy and Yaws Database System)
STEPS	STEPwise approach to non-communicable disease risk factor surveillance
TAS	Transmission Assessment Survey
TCPS	Treponema Control Programme Simplified
TPHA	*Treponema pallidum* haemagglutination assay
WHO	World Health Organization
