# Supplementary material for: Challenges to ‘Last Mile’ Surveillance: Result of Programmatic Review of Integrated Skin NTDs Surveillance in Three Indonesian Districts
Source: Trop Med Infect Dis. 2026 May 6;11(5):123. doi: 10.3390/tropicalmed11050123 (PMC13211722; doi:10.3390/tropicalmed11050123)
Supplement: Supplementary file 1 [file tropicalmed-11-00123-s001.zip › tropicalmed-4101004-supplementary.pdf]

The table below presents the reported disease elimination progress for yaws, leprosy, and lymphatic filariasis in Indonesia over the recent reporting period. The data provide a consolidated overview of disease trends and the current epidemiological situation at the national level. Overall, the low prevalence rates and progressive reductions in transmission reflect Indonesia's sustained efforts and progressive achievements in moving towards the 2030 elimination targets. These figures illustrate that the country is entering the 'last mile' of elimination, where intensified surveillance, targeted interventions, and strong programme stewardship are critical to securing and sustaining interruption of transmission.

**Table S1.** Detailed disease elimination targets and progresses of Indonesia.

| Diseases | Indicators | Baseline | Yearly Progresses |
| --- | --- | --- | --- |
| Leprosy | New leprosy cases with grade-2 disability (rate per 1,000,000 population) | 2014: rate of 6.2 per 1,000,000 population | 2015: 6.5 per 1,000,000<br>2016: 5.2 per 1,000,000<br>2017: 4.2 per 1,000,000<br>2018: 4.1 per 1,000,000<br>2019: 4.1 per 1,000,000<br>2020: 2.3 per 1,000,000<br>2021: 2.5 per 1,000,000<br>2022: 2.8 per 1,000,000<br>2023: 3.0 per 1,000,000<br>2024: 4.0 per 1,000,000 |
| | New leprosy child case (<15 years) detection rate per 1,000,000 population | 2014: rate of 26.4 per 1,000,000 with 17,025 new child cases | 2015: 26.8 (1,930 new child cases)<br>2016: 26.6 (1,923 new child cases)<br>2017: 24.2 (1,755 new child cases)<br>2018: 25.7 (1,861 new child cases)<br>2019: 28.3 (2,009 new child cases)<br>2020: 15.8 (1,126 new child cases)<br>2021: 16.2 (1,133 new child cases)<br>2022: 17.7 (1,226 new child cases)<br>2023: 17.0 (1,179 new child cases)<br>2024: 20.3 (1,420 new child cases) |
| | Number of new leprosy cases annually | 2014: 17,025 new cases with a prevalence of 0.79 per 10,000 population | 2015: 17,202 new cases (0.79 prevalence)<br>2016: 16,826 new cases (0.71 prevalence)<br>2017: 15,910 new cases (0.70 prevalence)<br>2018: 17,017 new cases (0.69 prevalence)<br>2019: 17,439 new cases (0.74 prevalence)<br>2020: 11,173 new cases (0.49 prevalence)<br>2021: 10,976 new cases (0.45 prevalence)<br>2022: 12,441 new cases (0.54 prevalence)<br>2023: 14,376 new cases (0.62 prevalence)<br>2024: 14,698 new cases (0.62 prevalence) |
| Yaws | Interruption of transmission for 3 consecutive years | 1952: prevalence rate of yaws of 4.11 per 100,000 population<br>1980: 0.45 per 100,000 population after the initiation of Treponema | Number of probable cases found:<br>2021: 149 cases (prevalence rate of 0.21)<br>2022: 68 cases (prevalence rate of 0.09)<br>2023: 79 cases (prevalence rate of 0.11)<br>2024: 181 cases (prevalence rate of 0.27) |

| | | | |
|---|---|---|---|
| | | Control Programme Simplified (TCPS) | |
| Lymphatic filariasis (LF) | Population requiring preventive chemotherapy (PC) for LF | 124 million people | 2021: 6,539,864 people<br>2022: 1,624,227 people<br>2023: 1,694,884 people<br>2024: 1,093,700 people |
| | Elimination as a public health problem through sustained infection below transmission assessment survey thresholds for at least 4 years after stopping MDA | 2014: 30 districts out of 236 endemic districts | 2015: 26 districts<br>2016: 23 districts<br>2017: 28 districts<br>2018: 38 districts<br>2019: 71 districts<br>2020: 68 districts<br>2021: 69 districts<br>2022: 77 districts<br>2023: 100 districts<br>2024: 128 districts |
